# Toxicity Profiles of Kleeb Bua Daeng Formula, a Traditional Thai Medicine, and Its Protective Effects on Memory Impairment in Animals

**DOI:** 10.3390/ph15080988

**Published:** 2022-08-11

**Authors:** Pornthip Waiwut, Kanchana Kengkoom, Wanassanun Pannangrong, Natdanai Musigavong, Chantha Chheng, Kusawadee Plekratoke, Pitchayakarn Taklomthong, Nutchareeporn Nillert, Supaporn Pitiporn, Pakakrong Kwankhao, Supawadee Daodee, Yaowared Chulikhit, Orawan Montakantirat, Chantana Boonyarat

**Affiliations:** 1 Faculty of Pharmaceutical Sciences, Ubon Ratchathani University, Ubon Ratchathani 34190, Thailand; 2 National Laboratory Animal Centre, Mahidol University, Nakorn Pathom 73170, Thailand; 3 Department of Anatomy, Faculty of Medicine, Khon Kaen University, Khon Kaen 40002, Thailand; 4 Center of Evidence-Based Thai Traditional and Herbal Medicine, Chao Phya Abhaibhubejhr Hospital, Mueang Prachinburi 25000, Thailand; 5 Faculty of Pharmaceutical Sciences, Khon Kean University, Khon Kean 40002, Thailand

**Keywords:** traditional herbal medicine, amyloid beta-induced memory deficit, sub-chronic toxicity, acute toxicity, *Nelumbo nucifera*, *Piper nigrum*, *Centella asiatica*

## Abstract

Kleeb Bua Daeng (KBD) formula has long been used in Thailand as a traditional herbal medicine for promoting brain health. Our recent reports illustrated that KBD demonstrates multiple modes of action against several targets in the pathological cascade of Alzheimer’s disease (AD). The main purpose of the present study was to determine the protective effect and mechanism of KBD in amyloid beta (Aβ)-induced AD rats and its toxicity profiles. Pretreatment with the KBD formula for 14 days significantly improved the short- and long-term memory performance of Aβ-induced AD rats as assessed by the Morris Water Maze (MWM) and object-recognition tests. KBD treatment increased the activities of the antioxidant enzymes catalase, superoxide dismutase, and glutathione peroxidase; reduced the malondialdehyde content, and; decreased the acetylcholinesterase activity in the rat brain. An acute toxicity test revealed that the maximum dose of 2000 mg/kg did not cause any mortality or symptoms of toxicity. An oral, subchronic toxicity assessment of KBD at doses of 125, 250, and 500 mg/kg body weight/day for 90 days showed no adverse effects on behavior, mortality, hematology, or serum biochemistry. Our investigations indicate that KBD is a nontoxic traditional medicine with good potential for the prevention and treatment of AD.

## 1. Introduction

Alzheimer’s disease (AD) is a type of dementia that causes deterioration in cognitive function as well as memory problems. Dementia currently affects around 57.4 million individuals throughout the world [1]. This population is expected to nearly double every 20 years and is therefore projected to reach 152·8 million by 2050 [1]. Confusion, anger, mood swings, long-term memory loss, and social disengagement are early-stage symptoms of AD. Memory and learning are controlled by the cholinergic system, which includes the hippocampus and cerebral cortex [2].

At present, there is no cure for AD; the drugs approved for treatment only delay AD onset by a few years. Multiple studies indicate that the aggregation of amyloid beta (Aβ) appears to act as a trigger for the other pathological pathways of AD, such as for tau hyperphosphorylation, reactive oxygen species (ROS) generation, inflammation, mitochondrial damage, and cholinergic system dysfunction [3,4,5,6,7,8]. The accumulation of senile plaques and soluble Aβ is associated with neurotoxicity [9,10]. The small, soluble Aβ can interact with the cell membrane and, consequently, disrupt its integrity [7,8,11,12]. These Aβ peptides penetrate and aggregate together within the membrane, span the bilayer, and create a pore, leading to uncontrolled Ca^2+^ influx [5,6]. The increase in Ca^2+^ ions can cause mitochondrial dysfunction, leading to increased production of ROS, changed membrane permeability, and neuronal apoptosis. A progressive decrease in neuronal activity is a well-known feature of AD [10] and there is evidence indicating that Aβ causes the inhibition of synaptic currents, and disruption of synaptic plasticity [10]. Oxidative stress has also been linked to AD development. ROS production is a result of cellular metabolism, and components of the cellular antioxidant defense such as catalase, glutathione peroxidase, hemoxygenase, and mitochondrial manganese (Mn) superoxide dismutase (SOD) play a crucial role in counterbalancing oxidative damage. There is evidence of free-radical overproduction in AD brain area in which Aβ is abundant [13]. Because of its high metabolic rate, the brain is particularly vulnerable to oxidative stress [14]. Additionally, this stress is correlated to Aβ, which itself can generate free radicals [15]. Aβ shows the ability to convert Cu^2+^ to Cu^+^. As such, the oxygen can interact with reduced metals and generate superoxide anions which, in turn, combine with two hydrogen atoms to produce hydrogen peroxide [3]. Then, the hydrogen peroxide may interact with other reduced metallic ions, forming hydroxy radicals through Fenton reactions. The overproduction of these ROS can cause oxidative damage such as lipid and protein peroxidation. Additionally, the radical form of Aβ can withdraw protons from the neighboring lipids or proteins, generating lipid peroxides and carbonyls, respectively [4].

One of the characteristic changes of the AD brain is the decrease of acetylcholine (ACh). ACh is responsible for the processing and storage of memories [16]. It is mostly found in the forebrain, cerebral cortex, and hippocampus areas, which are vulnerable to AD. Acetylcholinesterase (AChE) is responsible for ACh hydrolysis. Recent study indicated that AChE can promote the assembly of Aβ into fibrils [17]. In addition, some studies reported increased AChE activity within and around amyloid plaques. They demonstrated that Aβ peptides can stimulate AChE activity both in vitro and in vivo [18,19,20,21]. Therefore, these findings suggest that Aβ can increase AChE activity, and AChE increases the presence of amyloid plaques. In addition, high Ca^2+^ levels activate the mitogen-activated protein kinase (MAPK) pathway, which is involved in tau hyperphosphorylation. Microtubule-bound soluble tau supports axonal transport. Tau is hyperphosphorylated by the over-activity of the kinase enzyme, which may lead to the tau detachment and then microtubules disassembly. This then leads to the formation of soluble tau aggregates and insoluble paired helical filaments that ultimately form neurofibrillary tangles. Disassembly and instability of microtubules impairs axonal transport, and the direct toxic effects of soluble hyperphosphorylated tau and fibrillar tau may contribute to tau-mediated neurodegeneration and cell damage [22,23,24]. The result is neurotoxicity and cell death, especially in the cerebral cortex, suggesting that the cardinal pathogenesis of AD is the presence of amyloid plaques.

For centuries, people have used plants as an alternative medicine to treat and counteract memory impairments. Herbal formulations present chemical varieties and bioactivities. The complex interaction of herbal formulations might provide synergistic effects that might boost the therapeutic efficacy or lessen the negative effects of individual herbs. Among the medicinal plants, Kleeb Bua Daeng (KBD) formula has an extensive history of being used as a traditional herbal medicine; this formula has been prescribed in Chao Phya Abhaibhubejhr hospital, Thailand, as a brain tonic, sleep aid, and mood stabilizer since 2013 [25]. The KBD formula comprises parts of three different kinds of single herbal plants: *Nelumbo nucifera* Gaertn. (NN) petals, *Piper nigrum* L. (PN) fruits, and the aerial part of *Centella asiatica* (L.) Urb. (CA) mixed in a ratio of 1:1:1 (dry weight). Studies have described the effects of NN, PN, and CA in relation to AD pathogenesis [26,27,28]. In our recent study, we found that KBD has various mechanisms of action against multiple targets in the AD pathogenic cascade, including antioxidant, anti-AChE, anti-Aβ aggregation, neuroprotective, and anti-apoptosis activities. In addition, KBD improved both short- and long-term memory in amnesic mice with scopolamine-induced memory loss [29]. The daily administration of KBD significantly decreased unpredictable chronic mild stress (UCMS)-induced cognitive impairment in mice [30]. Thus, these findings are supportive of protective effects of KBD based on its action against multiple targets related to AD and in improving cognitive impairment. Each active ingredient of KBD has been studied for their individual safety and toxicity, but data on the combination of the three herbs (NN, PN, and CA) are limited. Therefore, in this study, we investigated the protective effect of KBD on memory impairment in the Aβ-induced rat model and its mechanism and evaluated the acute and subacute toxicities resulting from oral administration of KBD.

## 2. Results

### 2.1. KBD Does Not Induce Cytotoxicity in Human Neuroblastoma Cells (SH-SY5Y)

**Figure 1:**
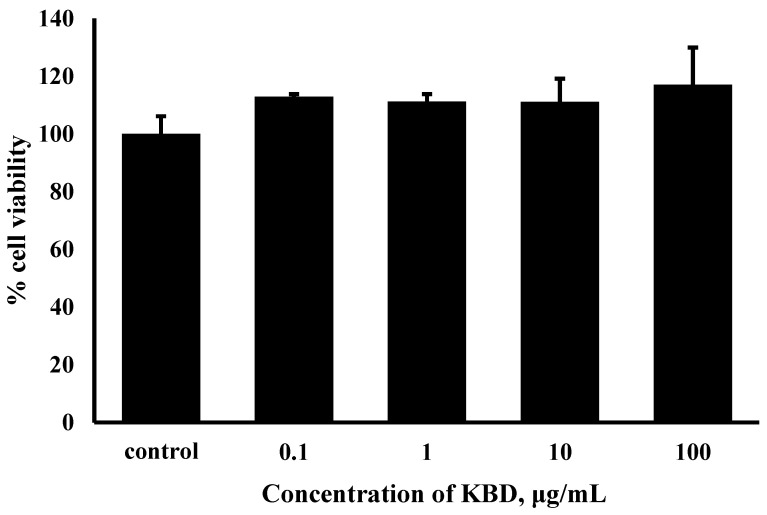
Effects of KBD on the viability of SH-SY5Y cells. The values are reported as mean ± SD (*n* = 4); One-way ANOVA was used to compare between control and test inhibitors.

We first checked whether KBD causes cytotoxicity in human neuroblastoma SH-SY5Y cells. To measure the cytotoxicity of KBD, SH-SY5Y cells were treated with various concentrations of KBD (0.1, 1, 10, and 100 μg/mL) for 24 h and the cell viabilities were then evaluated by MTT assay. KBD treatment did not induce cytotoxicity at concentrations of 0.1 to 100 μg/mL (Figure 1).

### 2.2. KBD Reduced Neuronal Cell Death Induced by Aβ_1–42_

**Figure 2:**
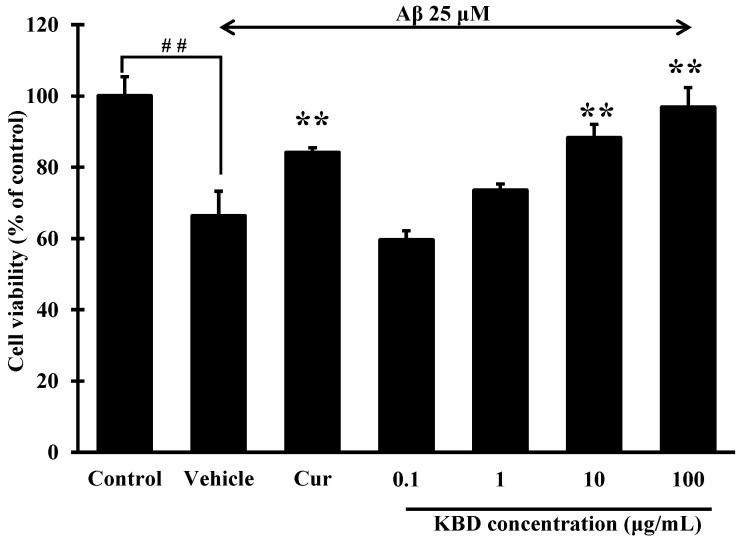
Effects of KBD at concentrations of 0.1 to 100 μg/mL on Aβ_1–42_-induced cell death in SH-SY5Y cells. Curcumin (Cur) at 10 μM was used as a reference standard. The values are reported as mean ± SD *(n* = 4). One-way ANOVA followed by the Tukey test, ** *p* < 0.01 versus Aβ_1–42_-treated group and ^##^ *p* < 0.01 versus the control group.

Aβ-induced neuronal-cell death is considered a critical event in the pathogenesis of AD. As estimated by MTT assay (Figure 2), after a 24 h exposure to Aβ_1–42_ 25 μM, the viabilities of SH-SY5Y cells significantly decreased to 66.31% compared with the control groups. When the cells were pretreated with KBD at various concentrations (0.1, 1, 10, and 100 μg/mL) for 24 h, and then exposed to Aβ_1–42_ for 24 h, SH-SY5Y cell viability significantly increased at doses of 10 and 100 μg/mL compared to the group only treated with Aβ_1–42_.

### 2.3. KBD Does Not Affect Locomotor Function in Rats

**Figure 3:**
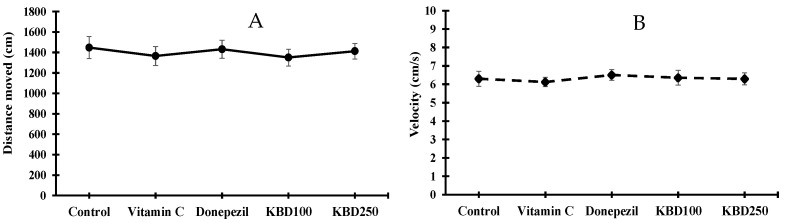
Average distance moved (**A**) and velocity (**B**) of rats in open field test. Data are expressed as mean ± SEM (*n* = 10). KBD100: KBD at a dose of 100 mg/kg/day; KBD250: KBD at a dose of 250 mg/kg/day. Vitamin C at a dose of 200 mg/kg/day and donepezil at a dose of 1 mg/kg/day were used as standard references.

After the administration of drugs (KBD at a dose of 100 and 250 mg/kg/day) and standard reference compounds (vitamin C at a dose of 200 mg/kg/day and donepezil at a dose of 1 mg/kg/day) for 2 weeks, the effect of the test drugs on locomotor function was evaluated by open-field test (OFT). The spontaneous locomotor activity is usually expressed as the velocity and distance that the rat moves in the OFT apparatus for 5 min. All rats were observed for motor ability. We found no significant difference in average velocity or distance moved amongst all groups, indicating that none of the treatments influenced locomotor function (Figure 3).

### 2.4. KBD Protects against Memory Loss in Aβ_1–42_-Treated Rats

In the in vitro study, KBD reduced the neurotoxicity induced by Aβ_1–42_. KBD also reduced the oxidative stress induced by H_2_O_2_ in our previous study [29]. Here, we investigated whether KBD is therapeutically effective in AD rats induced by Aβ_1–42_. The protective effects of KBD (100 and 250 mg/kg, p.o.) and standard reference compounds, vitamin C (200 mg/kg, p.o.) and donepezil (1 mg/kg, p.o.), on learning and memory impairment in rat induced by Aβ_1–42_ were investigated using the Morris Water Maze and object recognition tests.

#### 2.4.1. Water Maze Test (MWM) 

**Figure 4:**
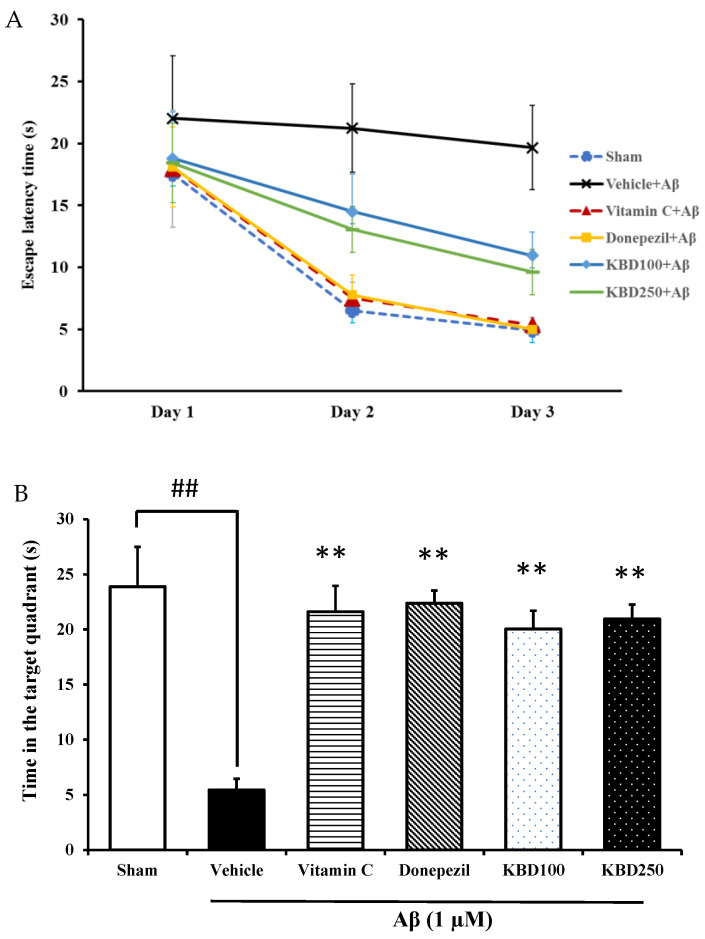
The effect of KBD formula on memory impairment induced by Aβ_1–42_ in the MWM test: (**A**) escape latency phase; (**B**) time spent in the target quadrant in probe test phase. The data are shown as mean ± SEM (*n* = 8). ^##^, ** *p* < 0.01 showed a significant difference compared with control and Aβ groups, respectively. Vitamin C at 200 mg/kg and donepezil at 1 mg/kg were used as standard references.

The MWM test was conducted to study hippocampal-dependent spatial-learning ability. In the training trials, the escape latency of the rats treated with Aβ_1–42_ was significantly higher compared with the sham group, whereas KBD-treated rats showed improvements compared with the Aβ-induced rats (Figure 4A). In the probe trial, the platform was removed. KBD-treated rats spent significantly more time in the target quadrant than the Aβ-induced rats (Figure 4B). The positive control rats, those treated with the standard reference compounds vitamin C 200 mg/kg or donepezil 1 mg/kg, required less time to find the platform in the training trials and spent more time swimming in the target quadrant compared with the Aβ-induced rats. Thus, these results demonstrate that KBD treatment significantly protected against spatial memory loss in Aβ_1–42_-treated rats.

#### 2.4.2. Object-Recognition Test (ORT)

**Figure 5:**
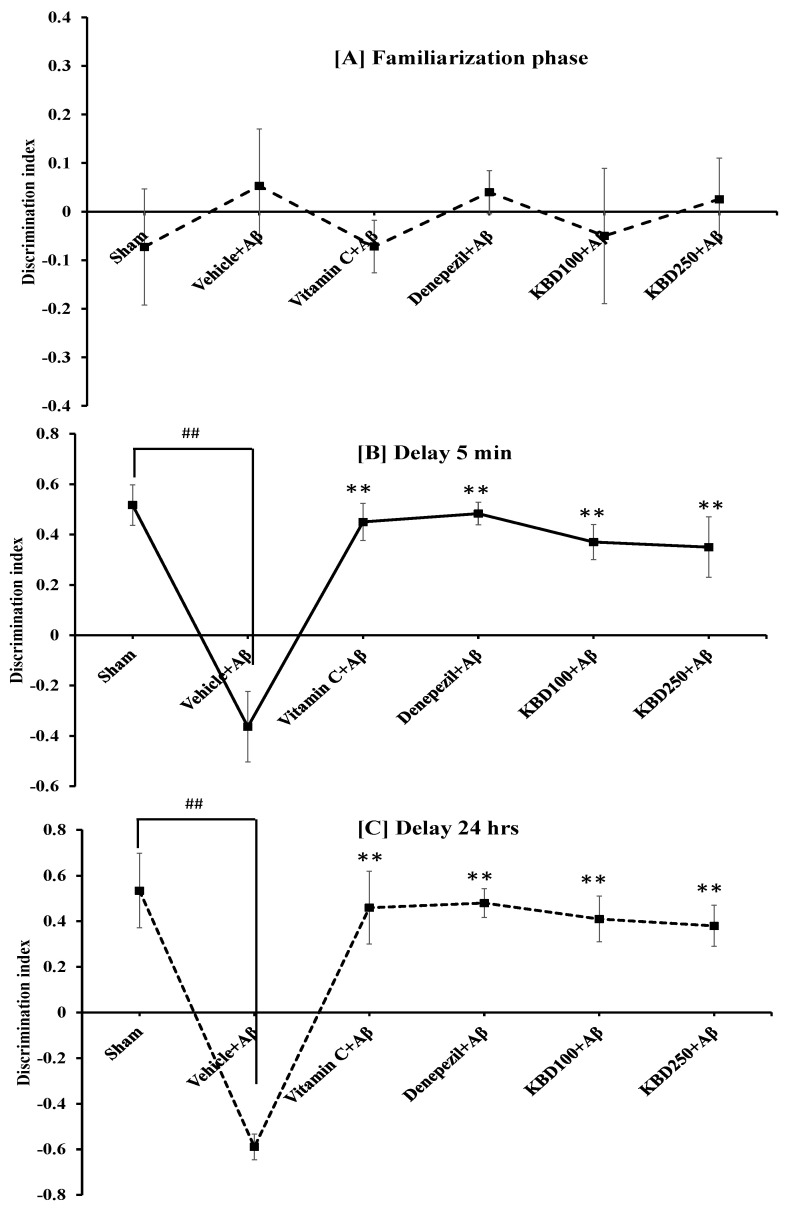
Effect of KBD formula on memory impairment induced by Aβ_1–42_ (1 µM) in the ORT test: (**A**) the familiarization phase; (**B**) 5-min delay test; (**C**) 24-h delay test. The data are shown as mean ± SEM (*n* = 8). ^##^ *p* < 0.01, between control and β-amyloid group; ** *p* < 0.01, between β-amyloid group and treatment group. Vitamin C at 200 mg/kg/day and donepezil at 1 mg/kg/day were used as standard references.

Recognition memory was evaluated by ORT after the administration of Aβ for 14 days. In the training session, we observed no significant difference in the familiarization phase between all groups of animals because both objects were novel (Figure 5A). However, in the 5-min delay phase (Figure 5B), all group of animals explored the novel object significantly more than the familiar object, indicating recognition memory retrieval, with the exception of the Aβ_1–42_ group. Moreover, in the long-term retention 24-h delay test (Figure 5C), rats treated KBD formula at doses of 100 and 250 mg/kg BW tended to have a higher discrimination index (DI), indicating they spent significantly more time exploring the novel object than those in the Aβ-treated group. The group administered Aβ_1–42_ showed significant memory impairment compared with the control group (*p* < 0.01).

### 2.5. KBD Reduces Malondialdehyde (MDA) Levels in Rat Brain

**Figure 6:**
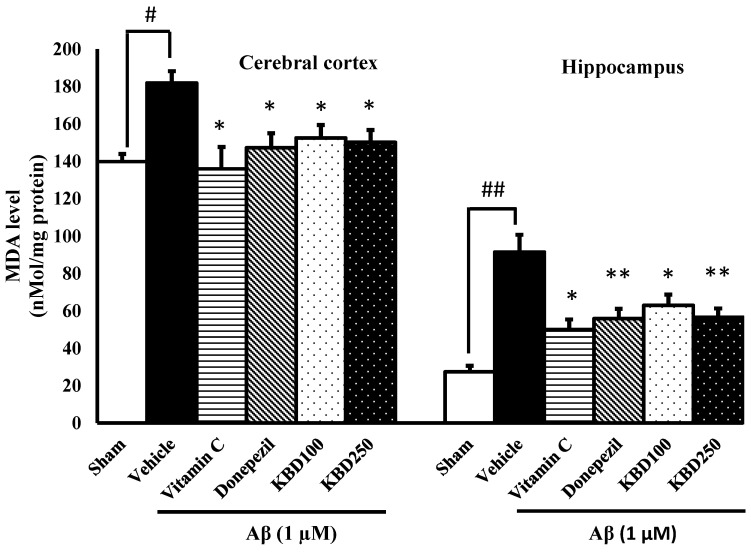
Effects of KBD on the cerebral cortex and hippocampal MDA levels. Values are mean ± SEM (*n* = 4). Statistical significance was determined by one-way ANOVA following with Turkey’s post hoc analyses. ^#^ *p* < 0.05 and ^##^ *p* < 0.01, significant difference compared with sham group. * *p* < 0.05 and ** *p* < 0.01, significant difference compared with Aβ group.

To further characterize the ability of KBD to inhibit oxidative stress in vivo, changes of MDA levels were analyzed after behavioral tests. MDA is an important biomarker of lipid peroxidation and high MDA levels are reported in an animal model of amyloidosis in Alzheimer’s [31]. The administration of Aβ_1–42_ significantly elevated the level of MDA in the hippocampal and cortex regions (Figure 6). The MDA levels in the hippocampus and cerebral cortex of Aβ_1–42_-treated rats increased to 91.48 ± 9.23 and 177.28 ± 6.90 nmol/mg protein, respectively, compared with the sham group (27.42 ± 3.24 and 144.66 ± 5.83 nmol/mg protein, respectively). KBD pretreatment (100 and 250 mg/kg, p.o.) prevented this increase in MDA content induced by Aβ_1–42_ in the hippocampal and cortex regions.

### 2.6. KBD Enhances Oxidative Enzymes Activity in Rat Brain

**Figure 7:**
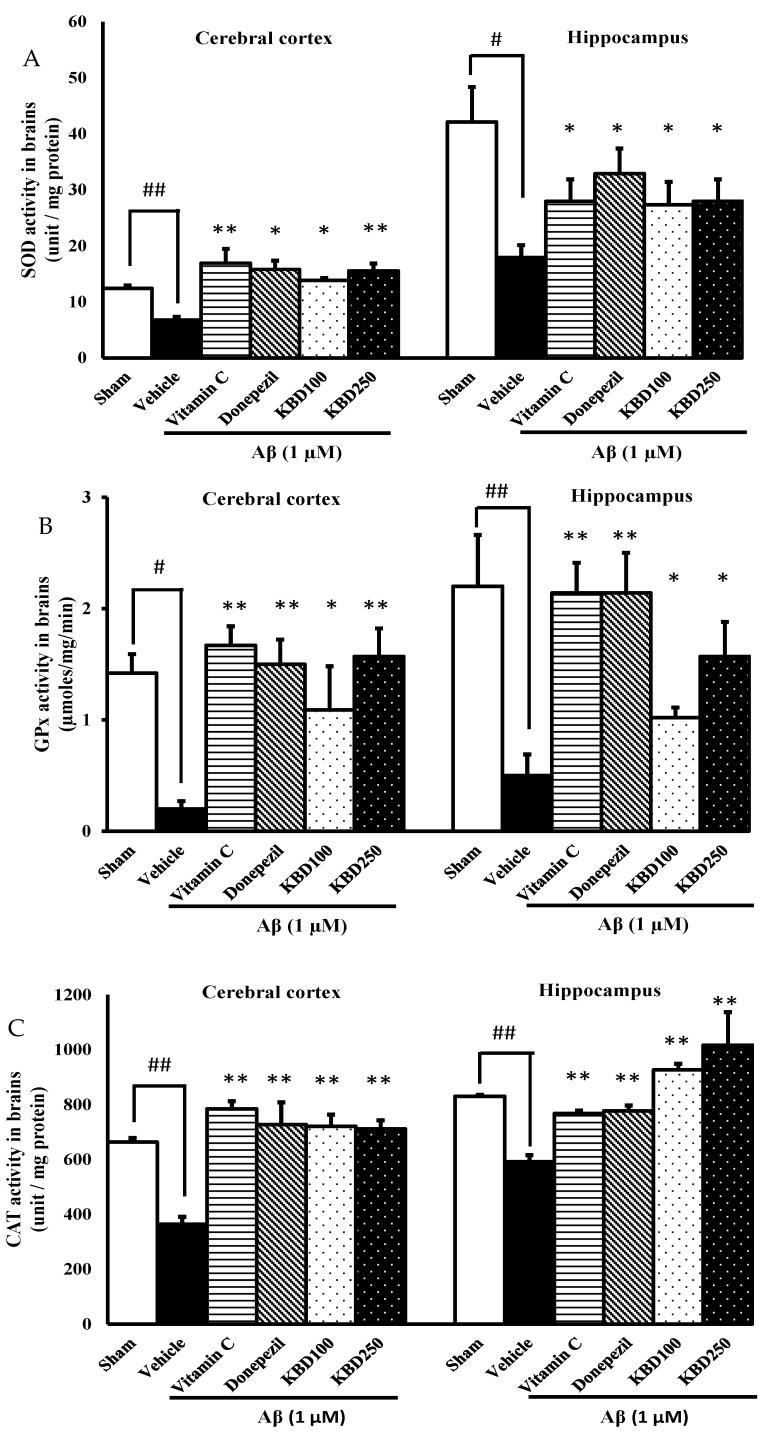
Effects of KBD on oxidative enzymes activity in the cerebral cortex and hippocampus; (**A**): superoxide dismutase (SOD), (**B**): glutathione peroxidase (GPx), (**C**): catalase (CAT). Values are mean ± SEM (*n* = 4). Statistical significance was determined by one-way ANOVA following with Turkey’s post hoc analyses. ^#^ *p* < 0.05 and ^##^ *p* < 0.01, significant difference compared with sham group. * *p* < 0.05 and ** *p* < 0.01, significant difference compared with Aβ group.

In the brain, superoxide dismutase (SOD), catalase (CAT), and glutathione peroxidase (GPX) are the major antioxidant enzymes responsible for scavenging free radicals generated in oxidative stress process. In comparison with the vehicle group, injection with Aβ_1–42_ resulted in significant decreases in SOD (Figure 7A), GPX (Figure 7B), and CAT (Figure 7C) specific activities. Pretreatment with KBD 24 h before exposure to Aβ_1–42_ showed protection against oxidative stress in hippocampal and cortex regions through elevated specific SOD, CAT, and GPX activities in comparison with Aβ_1–42_ rats.

### 2.7. KBD Alters the Level of AChE Activity in Rat Brain

**Figure 8:**
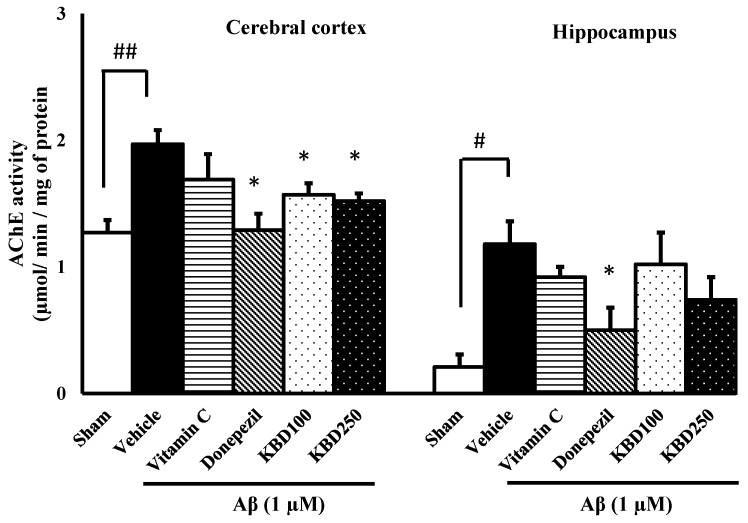
The effect of KBD on AChE activities in the brains of β-amyloid-induced rats. The data are shown as mean ± SEM. ^#^ *p* < 0.05 and ^##^ *p* < 0.01 compared with control; * *p* < 0.05 compared with Aβ groups (*n* = 4).

The effects of the herbal formula on the AChE function were investigated in the hippocampus and in cerebral cortex tissues of rats induced by Aβ_1–42_ and the results were represented in Figure 8. The results showed that AChE activity was significantly increased in the rat group exposed to Aβ_1–42_, compared to the control group. The rats pretreated with donepezil presented a significant decrease in AChE activity for both cortex and hippocampus tissues compared to the Aβ-treated group, while the vitamin C-treated group showed no significant difference to the Aβ group. Pretreatment with KBD at the concentrations of 100 and 250 mg/kg/day before exposure to Aβ resulted in the reduction of AChE activity in the cortex, but not in the hippocampus.

### 2.8. Acute and Subchronic Toxicological Evaluation

#### 2.8.1. Acute Toxicological Evaluation

All the rats treated with KBD at concentrations of 300 and 2000 mg/kg were alive for all 14 days of observation. At a dose of 2000 mg/kg, KBD produced no treatment-related signs of toxicity in any of the animals over 14 days. Normal body weight gains were observed in all of the dose groups. In addition, no abnormal gross findings were observed in any of the animals. The oral acute toxicity of KBD capsule powder (LD_50_) was therefore considered to be unclassified since none of the tested doses, up to 2000 mg/kg, induced death or toxic symptoms.

#### 2.8.2. Subchronic Toxicological Evaluation

##### Survival and Clinical Observations

No treatment-related mortality was observed in any of treatment groups during administration of KBD or when it was withdrawn for 14 days. The behaviors of rats were not adversely affected by KBD treatment (groups II–V). The rats appeared healthy and no notable signs of toxicity were observed throughout the experimental period.

##### Body Weight and Food Consumption

**Figure 9:**
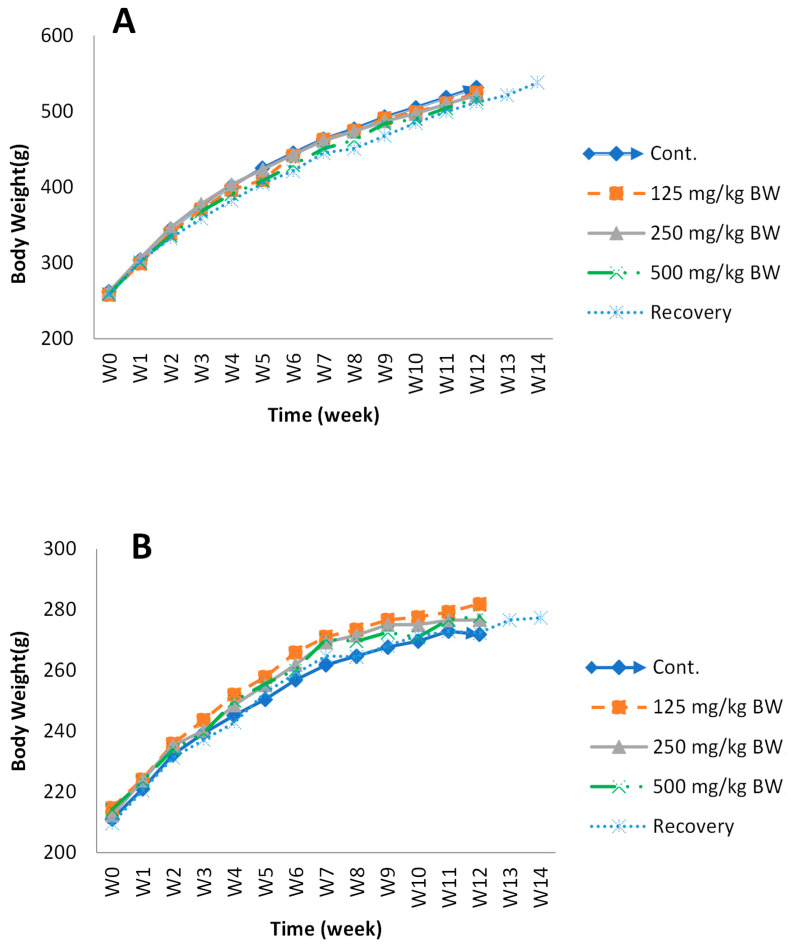
The body-weight change in male (**A**) and female (**B**) rats during the administration of KBD and during the recovery period. Group I, control group (vehicle); Group II, low-dose KBD group (125 mg/kg BW); Group III, medium-dose KBD group (250 mg/kg BW); Group IV, high-dose KBD group (500 mg/kg BW); Group V, recovery group (500 mg/kg BW). Each point represents mean ± SEM, *n* = 10.

In the subchronic toxicity study, the body weight gain of all groups of rats was sustained throughout growth. The results are shown in Figure 9. When compared with the control group, the body weight of rats in the KBD groups showed no significant differences (*p* > 0.05) during the experimental period. Average food consumption per day was approximately 21.1–21.8 g/day in males and 12.4–13.8 g/day in females.

##### Hematology Parameters

The hematology parameters were analyzed at the end of the 90-day treatment and recovery period (14 days). The results of the hematological study are shown in Table 1. From the evaluation of hematological parameters, we observed an increase in platelet levels in the female groups treated with a high dose of KBD (500 mg/kg and recovery groups). No significant differences were observed between the vehicle control and KBD treatment groups in the other hematological parameters such as RBC, HGB, HCT, and WBC.

##### Serum Biochemical Parameters

Changes in the serum biochemical parameters are shown in Table 2. In male rats, AST, ALT, and GLU in the recovery group were significantly higher than in the control group and other treatment groups. In female rats, TG was significantly increased in the 500 mg/kg group compared with the control group. Meanwhile, a decrease in the CREA level was observed. Although both sexes showed significant differences in some parameters compared with the control, all biochemistry parameters were in the normal range.

##### The Relative Organ Weight

After KBD treatment for 90 days, thirteen organs (brain, thymus, heart, lung, liver, spleen, kidneys, adrenal glands, testis, epididymis, ovaries, and uterus) were isolated and weighed at necropsy (Table 3). The relative organ weights of all organs in both sexes were in the normal level. No significant differences were observed between the control group and KBD-treated groups, except the epididymis of recovery group, which showed a lower relative weight compared with the control group.

##### Histopathological Analysis 

**Figure 10:**
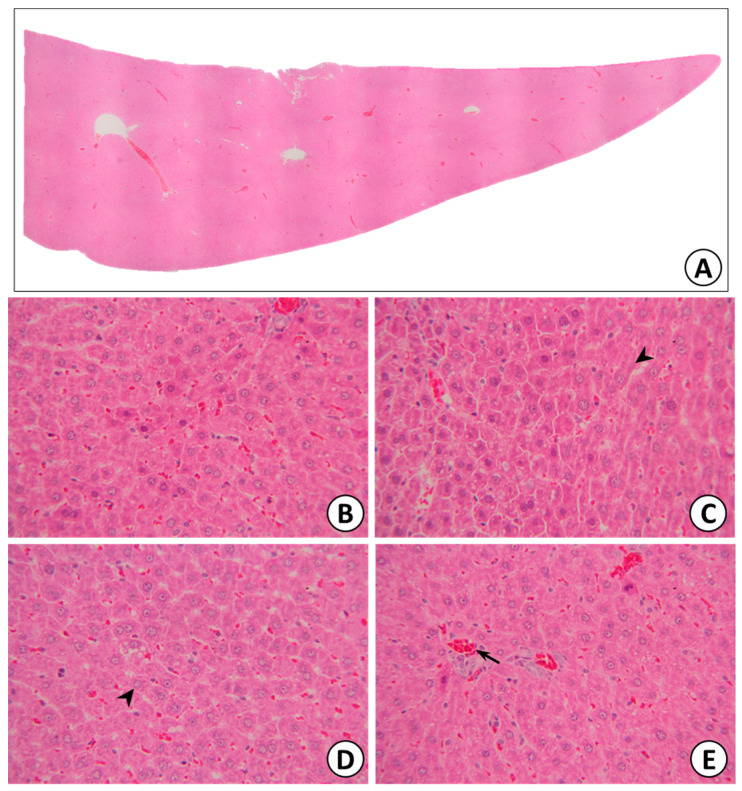
Liver (**A**), normal hepatic lobules (**B**), normal hepatocytes with radiate plate arrangement in hepatic lobules (black arrow: hepatocyte) (**C**,**D**), and normal central vein in hepatic lobules (black arrow: central vein) (**E**).

The liver was sectioned and stained for histopathological examination. No changes were observed in any of the parameters evaluated in the liver (Figure 10), such as hepatocyte regeneration/degeneration, architectural distortion, necrosis, interface hepatitis, portal infiltration, central venous hemolysis, or obstruction/dilatation of the bile duct.

## 3. Discussion

Due to the multiple pathways involved in AD pathogenesis, the classical single-target approach that modulates one target has been proven to be ineffective. Kleeb Bua Daeng (KBD) formula is a traditional Thai medicine that comprises three herbal plants: *Nelumbo nucifera* Gaertn. (NN) petals, *Piper nigrum* L. (PN) fruits, and the aerial part of *Centella asiatica* (L.) Urb. (CA) mixed in a ratio of 1:1:1 (dry weight). Our recent reports illustrate that KBD demonstrates multiple modes of action against multiple targets in the AD pathological cascade, including inhibiting AChE, scavenging free radicals, inhibiting amyloid aggregation, neuroprotection, and the improvement of cognitive impairment induced by chronic mild stress in mice [29,30]. The main purpose of the present study was to determine the protective effect and mechanism of KBD in Aβ_1–42_-induced AD rats and its toxicity profiles, including acute and subchronic toxicities. Here, we investigated whether KBD was therapeutically effective in Aβ_1–42_-induced AD rats. The protective effects of KBD (100 and 250 mg/kg, p.o.) and standard reference compounds, including an antioxidant (vitamin C, 200 mg/kg, p.o.), and acetylcholinesterase inhibitor (donepezil,1 mg/kg, p.o.) on learning and memory impairment in Aβ_1–42_-induced AD rats were investigated using the MWM test and ORT.

The accumulation of Aβ plaques is a key hallmark of AD. Amyloid plaques are toxic to neurons and lead to the impairment of learning and memory in rats. Thus, rats with memory deficits induced by Aβ can be used as an animal model for AD [32]. Prior studies have revealed that Aβ_1–42_ can persist in hippocampal tissue for weeks following direct administration into the brain via the intracerebroventricular method and can impair memory and learning function [33]. The presence of Aβ leads to the oxidative modification and degeneration of neurons. Previous studies have also indicated that Aβ can act as a neurotoxin via mechanisms involving the generation of hydrogen peroxide and lipid peroxidation [34]. Thus, in the present study, Aβ_1–42_ was chosen to induce memory impairment in rats.

In healthy rats, after drug administration for 2 weeks, the average velocity and total distance moved for the rats treated with KBD during the OFT showed no statistically significant differences among the groups, which indicated that KBD did not affect locomotor function. After 2 weeks of Aβ_1–42_ injection, the performance of the Aβ_1–42_-treated rats in both the water maze and novel object-recognition tasks was significantly impaired compared to that in the sham group. These results are in line with those of a previous study by Zhu et al., who reported a decrease in learning and memory ability in both short- and long-term tasks in Aβ-treated rats [35].

Recognition memory depends on the ability of rats to discriminate new and familiar objects. An enhanced percentage discrimination index can indicate that a treatment protects against cognitive deficits. The object-recognition test has been used in many studies as an appropriate model for assessing learning and memory in rats because it relies on the natural novelty and curiosity behavior of rodents [36].

In the first test period (5 min delay), the rats in all the groups, except the Aβ_1–42_ group, showed a preference for the novel object, which was observed as an increase in the amount of time spent on the new object. The Aβ_1–42_ group failed to discriminate between new and familiar objects, indicating that the Aβ_1–42_ injected rats had dysfunctional non-spatial working memory. We found that KBD at doses of 100 and 250 mg/kg BW could ameliorate the cognitive impairments in short-term memory.

The same outcome pattern was also found in the object recognition test after a 24-h delay. These results indicate a significant improvement in long-term memory in all the treated groups. Donepezil is a well-established standard treatment for AD. In this study, donepezil was used as a positive control. Many studies have demonstrated that donepezil can inhibit Aβ plaque deposition and prevent synapse loss in mouse models of AD [37,38]. Here, the effect of KBD was comparable to that of donepezil. Vitamin C at 200 mg/kg BW was used as an additional positive control. Although the mechanism by which vitamin C impacts brain pathology in AD or animal models is unclear, a study by Kook et al. revealed that high-dose oral supplementation of vitamin C could reduce amyloid plaques in the cortices and hippocampi of transgenic mice [39]. Recently, Murakami and coworkers reported that vitamin C could ameliorate the impairment of memory and learning in Aβ-injected mice [40]. This scientific evidence supports the use of donepezil and vitamin C as reliable reference standards.

The Morris Water Maze test was applied to investigate the spatial learning and long- term memory of Aβ-induced rats [41]. In the water maze test, a longer swimming time in the target quadrant demonstrates better spatial learning and long-term memory. The time in the target quadrant was significantly enhanced for rats pretreated with KBD compared to that for the group treated with only Aβ_1–42_. This outcome indicated improvement in spatial memory. The present study is the first to describe the efficacy of KBD for preserving learning and memory performance in Aβ-treated rats.

It has been reported that Aβ deposits are related to several biomarkers of oxidative stress including lipid peroxidation, SOD, GPx and CAT [42,43,44,45]. Butterfiels et al. [46] reported that large accumulations of Aβ_1–40_ and Aβ_1–42_ were related to increased protein oxidation and lipid peroxidation in the cortex and the hippocampus in AD. Additionally, brain regions with low levels of Aβ, such as the cerebellum, do not show high concentrations of oxidative stress markers [47,48,49]. MDA is a common aldehyde that is generated from the lipid peroxidation, making it an appropriate representative of oxidative stress [50]. One enzymatic pathway in particular has been reported to play a role in the scavenging of ROS and other free radicals. In this pathway, SOD first performs a catalytic function, converting superoxide anions to hydrogen peroxide. Next, CAT and GPx remove the toxic hydroxyl radicals formed by hydrogen peroxide [51]. An increase in SOD activity may result in the excess production of hydrogen peroxide, which must be neutralized by CAT and GPx [52]. Some clinical trials have reported that AChE inhibitors influence the oxidative balance in AD patients [53]. Thus, the evaluation of potential drugs for AD should focus on those that perform well in increasing the activity of antioxidant enzymes while decreasing lipid peroxidation and lowering MDA levels. Here, we found significantly impaired CAT, GPx and SOD activities in hippocampi and cerebral cortices of Aβ groups. Likewise, Aβ promoted an increase in lipid peroxidation. These findings indicate the cytological effects of oxidative stress induced by Aβ_1–42_ and are consistent with a previous study by Jhoo et al. [54]. Rats treated with KBD at doses of 100 and 250 mg/kg/day showed significant increases of CAT, GPx and SOD activities in cortical and hippocampal tissues compared to the Aβ_1–42_ group. A significant reduction in MDA levels in the cortical and hippocampal tissues was also observed in rats pretreated with KBD at concentrations of 100 and 250 mg/mL compared to the Aβ group. Rats treated with KBD showed comparable results to the donepezil and vitamin C groups, indicating that KBD potentially boosted the antioxidant capacity in Aβ-induced rats by increasing the activity of endogenous antioxidative enzymes that act against oxidative stress. Our findings indicated that KBD could stimulate antioxidant enzyme activity and decrease lipid peroxidation in brain tissue. These positive results could be due to the numerous phytochemical compounds found in the KBD formula. Our previous study suggested that KBD ameliorated cognitive impairment in unpredictable, chronic, mild, stress-induced rat via the improvement of antioxidant capacity of the flavonoid, phenolic acid, and triterpene glycosides, which are constituents of the plant ingredients [30]. We recently analyzed the phytochemicals in the KBD formulation and found that the herbal medicine was rich in asiaticoside and madecassoside (major active constituents of CA), piperine (a major component of PN), kaempferol, quercetin, rutin, kaemferol-3-glucoside, luteolin-7-O-glucoside, and ferulic acid (major constituents of NN) [30,55]. The major phytochemicals found in the KBD formula were triterpene glycosides—madecassoside (179 mg/g ex-tract) and asiaticoside (57 mg/g extract)—and alkaloid piperine (10 mg/g extract). The antioxidant activities of these compounds have been extensively reported [56,57,58,59,60,61,62]. Bian et al. suggested that madecassoside could counteract the increase in MDA levels caused by hydrogen peroxide. Madecassoside inhibits apoptosis by downregulating the activation of caspase-3 and p38 MAPK and protecting mitochondrial membranes [56]. In another study, the protective effect of asiaticoside was tested in hippocampal slices and cell cultures; it was shown to be effective at lowering the level of intracellular free radicals and reducing the cell death caused by hydrogen peroxide [57]. Kaempferol increases the levels of antioxidant enzymes in the brain, including SOD and glutathione. Previous research on kaempferol in a rat cancer model found that the compound had rejuvenating activities due to the activity of the antioxidant enzymes CAT, SOD, and GPx [58]. In addition, kaempferol was shown to reduce the levels of MDA and tumor necrosis factor-α [59]. The administration of piperine led to significantly increased SOD, CAT, and GPx activities in the hearts, livers, kidneys, aortas, and intestines of rats fed high-fat diets, indicating reduced oxidative stress in cells [60]. Flavonoids, such as luteolin-7-O-glucoside, can protect against the cognitive impairment induced by oxidative stress [61,62]. Taken together, based on the major phytochemicals found in the KBD formula and the activities of the chemical constituents, the lipid peroxidation = inhibitory activity of the KBD is likely to be provided by triterpene glycosides madecassoside and asiaticoside, while the activity enhancing antioxidant enzyme function mainly comes from piperine. In summary, it can be concluded that the oral administration of KBD at 100 and 250 mg/kg/day can defend neurons against oxidative stress in an animal model of Alzheimer’s disease by significantly decreasing MDA levels and enhancing endogenous antioxidant status.

Cognitive functions are associated with the cholinergic system, and abnormalities in this system have been found in AD. Acetylcholine is an important neurotransmitter, synthesized from the precursors acetyl CoA and choline. It is involved in several behavioral processes, including cognitive functions such as learning, attention, and memory [63]. The primary enzyme acetylcholinesterase can break down acetylcholine in the synaptic cleft, which can interfere with choline uptake and increase the hydrolysis of acetylcholine. The activity of this enzyme can be blocked by cholinesterase inhibitors, which help the neurotransmitter to remain in the synaptic cleft longer. AD patients are often prescribed cholinesterase inhibitors in the early stages of disease [64]. In the present study, the effect of the herbal formula on AChE function in the brain tissues of Aβ-induced rats was investigated. We observed the highest activity of AChE in Aβ-treated rats, which is consistent with a study by Carson et al. regarding the association between Aβ plaques and AChE [65]. AChE was increased in the brains of rats that received intra-cerebral-ventricular injections of the Aβ peptide. The herbal formula and vitamin C failed to significantly decrease the AChE activity in the hippocampus. Only donepezil produced a significant AChE-inhibitory effect in the hippocampus. However, a significant inhibitory effect was observed when using KBD at doses of 100 and 250 mg/kg in the cortical tissue. Kuhl et al. reported that AChE was often decreased in the cerebral cortex and the hippocampus [66]. One meta-analysis reported that a reduction in AChE activity of 41% was found in the cerebral cortex in AD postmortem examination [67]. The distribution of AChE activity throughout the different brain regions was dissimilar. A lower range of AChE activity was reported in hippocampal area, which was lower than that in the frontal cortical area [68]. The AChE activity in the cerebral cortex is mediated by acetylcholine transferase in cholinergic neurons [69]. This may support our results. A pharmacological mechanism has been reported for kaempferol, which has a strong positive effect in the treatment of central nervous system diseases. Kaempferol is a flavonoid found in many herbs, including *N. nucifera*. A kaempferol extract significantly inhibited acetylcholinesterase activity in brain tissue and prevented memory deficits in rats [70]. Quercetin found in the petals of *N. nucifera,* exerts a potential neuroprotective effect [71]. Quercetin administration protected against cognitive impairment in AD mice, reversing β-amyloidosis in the hippocampus and the amygdala [72]. However, these neuroprotective effects of quercetin are mainly associated with its antioxidant properties [73]. A methanolic extract of *C. asiatica* decreased the acetylcholinesterase activity in the hippocampi and cerebral cortices of scopolamine-induced amnestic rats due to the action of numerous triterpenes such as madecassoside, asiaticoside, madecassic acid, and asiatic acid [74]. To summarize, KBD exerts neuroprotective effects via a reduction in AChE activity, mainly in the cerebral cortical region. The major mechanism of KBD’s effects in AD treatment may be the reduction in AChE, similar to that for donepezil.

Taken together, our previous in vitro study, which showed that KBD demonstrates multiple modes of action against the AD pathological cascade including antioxidant, anti-AChE, anti-Aβ-aggregating, neuroprotective, and anti-apoptotic activities [29], and the present study, demonstrate that KBD can improve the short- and long-term memory performance of Aβ-induced AD rats by increasing the activities of the antioxidant enzymes CAT, SOD, and GPx; reducing the MDA content, and; decreasing the acetylcholinesterase activity in the brain. These activities result from the variety of phytochemical contents in plants that contain in KBD. Due to the multifactorial pathogenesis of AD, various classical agents have been proven to be ineffective because they have mostly been designed to target only one mode of action. KBD demonstrated a broader mode of action against AD compared to various classical agents such as tacrine, donepezil or vitamin C. Thus, KBD, which demonstrated multiple modes of actions, is likely to possess greater potential for AD treatment.

KBD has long been used as a traditional herbal medicine. However, its safety is often questioned by physicians and consumers. Hence, to provide safety information, the present study assessed the acute and subchronic toxicities of KBD using Wistar rats.

In the acute toxicity study, no treatment-related mortality occurred up to a dose of 2000 mg/kg BW. No obvious toxicological symptoms were detected when the rats were administered KBD at a dose of 2000 mg/kg BW. Thus, based on the toxicity classification [75], KBD was categorized as unclassified in the acute toxicity-hazard categories according to the Globally Harmonized Classification System (GHS). These results suggest that KBD is a relatively nontoxic drug [75].

For the subchronic toxicological evaluation, Wistar rats were administered various doses of KBD for 90 days—125, 250, and 500 mg/kg BW/day—based on the therapeutic doses used in clinics. The toxicological signs, mortality, body weight, water and food consumption, and hematological and biochemical parameters were assessed, and a pathological examination was carried out.

General behavior and body weight are critical parameters for the evaluation of toxicity. The body weight gain in all the groups of rats was sustained throughout growth. No significant difference was observed between the control group and the KBD-treated groups. Based on our clinical observations, all the rats appeared healthy, and no signs of toxicity were observed. These results indicate that KBD had no obvious influence on weight gain and did not cause behavioral changes in the rats, suggesting that KBD had no adverse effects on the growth of the rats.

After the administration of KBD for 90 days, the relative organ weight was assessed. Organ weight is a fundamental indicator of the functional status of animals. An increase in relative organ weight can indicate congestion, edema, or hypertrophy, whereas a decrease may indicate atrophy and other degenerative changes [76]. In this study, no significant differences in relative organ weight were observed between the experimental groups and the control group (*p* < 0.05).

Hematopoietic parameters are valuable for assessing the toxicity of drugs in humans and animals [77,78,79]. Treatment with KBD did not produce any statistically significant difference in hematological parameters, except for the platelet count, which was significantly higher in the high-dose and recovery groups of female rats compared to the control group. However, the PLT level was within the normal range. Hence, our results show that KBD had no effect on hematopoietic parameters.

With regard to biochemical parameters, the female 500 mg/kg group and recovery group showed significantly increased TG levels. These increases in TG were judged to be related to the treatment and were suggestive of hepatic damage; however, no changes in liver weight or histopathology were observed. Thus, this change was not considered to be treatment-related because the values were within the normal range and no abnormalities were detected with respect to the gross or histopathological examination of the livers. Additionally, no indicators of liver damage, such as ALT and AST were observed.

The liver is the main site of plasma protein synthesis [80], and any damage to the liver results in the consequent elevation of both ALT and AST in the blood. The determination of plasma protein parameters, such as the albumin, globulin, and albumin/globulin ratio, can provide criteria for assessing the synthetic capacity of the liver, since nearly all are synthesized in hepatocytes and a decrease in plasma proteins tends to reflect chronic damage [81]. The common pattern observed following significant hepatocellular damage is a reduction in albumin accompanied by a relative increase in globulins, which leads to a reduction in the albumin/globulin ratio, often with little change in the level of total protein. This pattern was observed in the male recovery group in the present study; however, no changes in liver weight or histopathology were detected, even in the high-dose group. Therefore, this finding was considered to be incidental. Thus, KBD did not cause liver toxicity in rats. This finding was consistent with our previous clinical study, which demonstrated no alteration in biochemical parameters of liver (serum AST, ALT) and renal functions (creatinine, and eGFR) in patients receiving KBD at a dose of 1000 mg twice a day for 3 months [82].

Overall results from our toxicities evaluation indicated that a high single dose administration of KBD (2000 mg/kg/day) did not show acute oral toxicity in rats. The oral subchronic toxicity assessment of KBD at doses of 125, 250 and 500 mg/kg body weight/day for 90 days revealed no adverse effects on behavior, mortality, hematology, serum biochemistry. No toxicopathologic lesions were detected following administration of high-dose KBD. KBD showed no toxicity on liver and kidney, which is consistent with our previous clinical study [82]. Moreover, our present results demonstrated that KBD had no adverse effects on the growth and hematopoietic parameters. Interestingly, toxicity was not observed even after doubling the clinical dose. Our finding may be useful in future studies as a guideline for dose adjustment of KBD.

## 4. Materials and Methods

### 4.1. Chemicals and Plant Preparation

The KBD formula powder was provided by Chao Phya Abhaibhubejhr Hospital Foundation, Prachinburi Province, Thailand. The relative herbarium voucher specimens of its plants components, (i) *Nelumbo nucifera* Gaertn., (ii) *Centella asiatica* (L.) Urb., and (iii) *Piper nigrum* L., were deposited at the museum of Chao Phya Abhaibhubejhr Hospital Foundation with the following voucher numbers: ABH15, ABH17, and ABH18, respectively. The plants were identified by Benjawan Leenin, the Head of the Traditional Knowledge Center, Chao Phya Abhaibhubejhr Hospital Foundation. The crude KBD extract was prepared by macerating the powders with ethanol at a 1:5 (*w*/*v*) ratio for 3 days, and this process was repeated twice. Concentrated extracts were obtained by filtration and then concentration using a rotary evaporator. The concentrated extracts were lyophilized into powder and stored in air-tight containers at 2–8 °C until use. The HPLC analysis of KBD has been reported in an earlier study by Maneenet et al. [30]. Acetylthiocholine iodide (ATCI), 5,5-dithio-bis-[2-nitrobenzoic acid] (DTNB), beta amyloid 1–42 (Aβ_1–42_), thiopental sodium, doneprezil, and vitamin C were purchased from Sigma Aldrich (SM Chemical supplies Co., Ltd., Bangkok, Thailand), Gibthai (GT Chemical supplies Co., Ltd., Bangkok, Thailand), and Fluka (SM Chemical supplies Co., Ltd., Bangkok, Thailand).

### 4.2. The Effects of KBD Formula on Aβ-Induced Cell Damage in Neuroblastoma Cells

#### 4.2.1. Cell Culture

Human neuroblastoma cells (SH-SY5Y) were maintained in Dulbecco’s modified eagle medium (DMEM/Ham’s F12) supplemented with 10% fetal bovine serum, 2 mM l-glutamine, 50 IU/mL penicillin, and 50 g/mL streptomycin at 37 °C in a humidified incubator containing 5% CO_2_.

#### 4.2.2. Cytotoxicity Assay

The cytotoxicity of KBD extracts was determined using a method previously described by [83]. SH-SY5Y cells (5 × 10^5^ cell/mL) were seeded into wells of a 96-well plate. After 48 h, the cells were treated with various concentrations of KBD extracts (0.1–100 μg/mL) for 24 h. Cell viability was then determined by incubating the cells with 3-(4,5-dimethyl-2-thiazolyl)-2,5-diphenyl-2H-tetrazolium bromide (MTT) (5 mg/mL in PBS) for 2 h. MTT solution was removed, and cells were lysed by DMSO. The absorbance was measured at 550 nm using a METERTECH Accureader M965 microplate reader. All data are expressed as a percentage of non-Aβ_1–42_-treated groups (control group). The cell viability of the control group is expressed as the proportion of 100%. The experiment was performed in independent triplicates (4 wells/group).

#### 4.2.3. Neuroprotection against Aβ_1–42_ Toxicity

The neuroprotective effect of KBD extracts was evaluated following a method previously described by [84]. The cells (5 × 10^5^ cell/mL) were plated in a 96-well plate and incubated for 48 h. Afterword, the cells were pre-incubated with various concentrations of KBD extracts (0.1–100 μg/mL) for 24 h and then were exposed to aggregated Aβ_1–42_ 25 μM, for 24 h. Cell viability was determined using the MTT colorimetric method. The absorbance of each well was measured at 550 nm with a METERTECH Accureader M965 microplate reader. Curcumin at the concentration of 10 μM was used as the reference standard. All data are expressed as a percentage of non-Aβ_1–42_-treated groups control group. The experiment was performed in independent triplicates (4 wells/group).

### 4.3. The Effects of KBD Formula on Aβ-Induced Memory Deficits in Rats

The effect of KBD formula on Aβ_1–42_-induced memory impairment in Sprague-Dawley rats was evaluated using two behavioral models: the Morris Water Maze (MWM) and the object recognition test (ORT).

#### 4.3.1. Animals Preparation

Healthy adult male Sprague-Dawley rats (6–8 weeks) weighing 240–300 g were purchased from Nomura Siam International Co., Ltd. The assessment was performed in accordance with the guidelines of the Animal Ethics Committee of Khon Kaen University (Approval No. IACUC-KKU-7/62). The animals were housed in plastic cages (5 rats/cage) and kept in a controlled environment at a temperature of 25 ± 2 °C, humidity of 50–55%, and a 12/12 h light/dark cycle (lights on from 6:00 a.m. to 6:00 p.m.) for one week before testing. Rats were allowed to free access to food and water.

#### 4.3.2. Drug Administration

**Figure 11:**
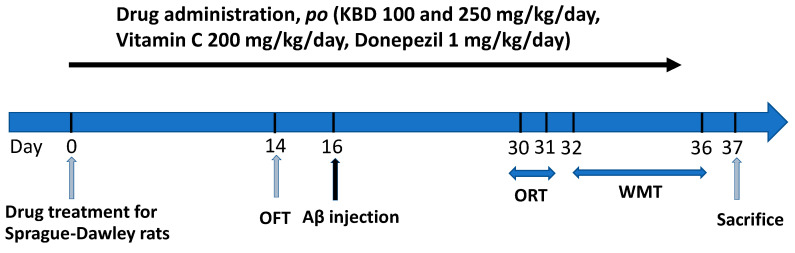
Animal experimental schedule.

Rats were randomly divided into six different groups (n = 8/group): first group, vehicle; second group, Aβ_1–42_; third and fourth groups, KBD treatment groups (100 and 250 mg/kg/day); fifth and sixth groups, positive control groups (donepezil 1 mg/kg/day and vitamin C 200 mg/kg/day). The KBD dose was calculated based on the clinical dose (2000–2400 mg/day) that was prescribed in the hospital [30]. This dose was converted into the appropriate dose for rats according to the following equation: human equivalent dose (HED, mg/kg) = rat dose (mg/kg) × (rat Km/human Km), where Km is a correction factor [85]. The KBD powder and reference standards were suspended in 0.5% carboxymethyl cellulose (CMC). Drugs were orally administered (p.o.) once daily until the end of experiment (Figure 11). After pretreatment with the KBD formula or reference standards for 14 days, the open-field test (OFT) was performed for locomotor function evaluation. At the day 16, the rats were injected with aggregated Aβ_1–42_ or vehicle by the bilateral ICV route and then treated with KBD or reference standards. Behavioral tests (water maze test (WMT) and object-recognition test (ORT)) were carried out 14 days after Aβ_1–42_ injection. All behaviors of the rats were tracked, observed, and recorded using a video camera. One day after the behavioral test, all rats were sacrificed for chemical analysis.

#### 4.3.3. Neurosurgery and Aβ_1–42_ Injection

To assess the effects of amyloid beta toxicity, Aβ_1–42_ (1 μM) was incubated at 37 °C for 24 h. Afterward, 1 μL of aggregated Aβ_1–42_ or normal saline (for sham-operated rats) was delivered into each lateral ventricle at a rate of 0.2 μL/min using a Hamilton microsyringe. Thiopental sodium was used to anesthetize male Sprague-Dawley rats, for which the operation was carried out using a stereotaxic device. The position of the stereotaxic referent points, planes, and the coordinate of bilateral ventricles were indicated on the skull diagram from the atlas of the rat brain in the stereotaxic coordinates of Paxinos and Watson [86]. Behavioral tests (water maze test and object recognition test) were carried out 14 days after neurosurgery.

#### 4.3.4. Open-Field Test (OFT)

The effect of KBD formula on rat locomotor function was assessed by an open-field test on the 14th day of chronic treatment with KBD formula. The open-field apparatus was a square box (50 cm × 50 cm × 40 cm) composed of polyvinyl chloride with black walls and a black floor. Each animal was placed individually into the center of the apparatus and allowed to explore freely for 5 min. During the test time, the distance and velocity traveled were recorded by digital camera and analyses were conducted using Noldus EthoVision XT version 12th software. The test apparatus was cleaned with a 75% ethanol solution after each individual animal test to remove any olfactory cues.

#### 4.3.5. Object-Recognition (ORT) Test

The object recognition test was carried out 14 days after Aβ_1–42_ injection (on the 30th day of drug treatment). ORT was performed to evaluate recognition memory. This task is based on the tendency of rodents to discriminate a familiar from a new object. The apparatus consisted of a square arena (50 cm × 50 cm × 40 cm) composed of polyvinyl chloride with black walls and a black floor. The objects to be recognized had visual patterns or visually different shapes that required discrimination. The ORT consisted of 3 phases: habituation, familiarization, and test phases. Rats were individually habituated to an open field box for 5 min. In the familiarization phase, after the habituation trial for 24 h, each rat was placed in the box for 5 min and exposed to two identical objects, A1 and A2. The time that rats explored each object was recorded. Test phase trials were performed 5 min and 24 h after the familiarization phase trial for short- and long-term memory assessment, respectively. In this trial, one of the two objects was replaced by a new object with different pattern or shape (B for 5 min delay or C for 24 h delay) in the original position. The rats were exposed to the two objects for 5 min and the total time spent exploring each of the two objects was measured. A discrimination index (DI) was calculated according to the following equation: DI = (Tn − Tf)/(Tf + Tn), where Tn and Tf represent the time spent to explore new and familiar objects, respectively. The box arena and objects were cleaned using 75% ethanol between trials to prevent a build-up of olfactory cues. The performance of the animals in this test was analyzed automatically via a SMART^®^ system.

#### 4.3.6. Morris Water Maze (MWM) Test

The water maze task [87] was conducted in a black circular pool 180 cm in diameter and 40 cm in height. The pool was filled with water (25 ± 1 °C) and divided into four quadrants (Q1–Q4) with a transparent platform submerged 2 cm below the water surface in the center of Q1. For the training trial, rats were allowed to search for the platform for 60 s. The time spent to reach the escape platform (escape latency time) was monitored. Each rat was trained through 4 trials per day for 3 consecutive days. For the probe test session, rats were allowed to swim from various quadrants in pool without the platform for 60 s. The swimming time in the target quadrant (Q1) where the platform had been placed was recorded.

### 4.4. Biochemical Parameter Assay

For the estimation of the biochemical parameters, after behavioral tests, the hippocampus and the cortex were precisely excised from the rat’s brains. The tissue samples were individually homogenized (1:10) in an ice-cold medium containing 0.04 M sodium phosphate buffer (pH 7.4) and finally centrifuged at 13,000× *g* at 4 °C for 10 min. The resulting supernatant was used for determination of specific superoxide dismutase (SOD), catalase (CAT), glutathione peroxidase (GPx) activities; acetylcholinesterase (AChE) activity, and malondialdehyde (MDA) content. The protein content of the brain supernatant was determined using bovine serum albumin as the standard [88].

#### 4.4.1. Determination of Superoxide Dismutase Activity

Superoxide dismutase (SOD) activity was measured using a method previously described by [89]. Briefly, a reaction cocktail was prepared by mixing 25 mL of 216 mM potassium phosphate buffer, 1 mL of 10.7 mM ethylenediaminetetraacetic acid (EDTA), 1 mL of 1.1 mM cytochrome C solution, and 50 mL of xanthine, and then adjusting the pH to 7.8. After, 900 μL of cocktail solution was combined with 50 μL of sample or standard. The reaction was initiated by adding 50 μL of xanthine oxidase enzyme (XOD) solution. The increase in absorbance at a wavelength of 540 nm was monitored for 5 min. The SOD activity in the sample was determined from the linear equation of the standard curve (units/mg protein).

#### 4.4.2. Determination of Catalase Activity

To assess the activity of the catalase (CAT), we followed the method previously described by [90]. Briefly, the reaction mixture, which contained 10 μL of supernatant and 60 μL of H_2_O_2_, was incubated at 37 °C for 1 min. The reaction was then stopped by the addition of 30 μL of 5 N sulfuric acid. The hydrogen peroxide concentration in the reaction was detected by the addition of 0.005 N of potassium permanganate solution. The absorbance of the reaction solution was measured at the wavelength of 515 nm. The CAT activity in the sample was determined from the linear equation of the standard curve. The data are expressed as unit/mg protein.

#### 4.4.3. Determination of Glutathione Peroxidase Activity

The glutathione peroxidase (GPX) activity was investigated based on the method described by [91]. Briefly, a cocktail solution was prepared by combining 23 mL of 1 mM sodium azide, 125 mL of 0.1 M glutathione, 0.5 mL of 6 mM β-NADPH, and 250 mL of 100 U/mL glutathione reductase. Supernatant (15 μL) was pre-incubated with 70 μL of cocktail solution and 15 mL of BTNB for 10 min at 37 °C in the dark. After, 15 μL of 4 mM H_2_O_2_ was added and incubated at 37 °C for a further 10 min in the dark. The decrease in absorbance was then measured at 415 nm. The enzyme activity to convert 1 μmol of NADPH to NADP in 1 min was defined as the activity of GPx, and the results are expressed as GPx units/mg protein. GPx levels were calculated from the standard curves constructed from the standard glutathione peroxidase.

#### 4.4.4. Determination of Malondialdehyde Level

For determination of the level of malondialdehyde (MDA), a marker of lipid peroxidation, we followed the procedure of [92]. A working solution of 1.5 mL of 0.05 M thiobarbituric acid and 1.5 mL of 20% acetic acid was added to 0.1 mL of supernatant, and the samples were heated at 95 °C for 1 h. After cooling, 5 mL of the mixture of n-butanol and pyridine (15:1, *v/v*) was added. The mixture was shaken vigorously. After centrifugation at 4000 rpm for 10 min, the absorbance of the organic layer was determined at the wavelength of 532 nm. The MDA value of the supernatant was determined by comparison with the standard curve. The results are expressed as nmol/mg protein.

#### 4.4.5. AChE Inhibitory Activity Assay

The KBD formula was investigated for its AChE inhibitory activity using a modification of Ellman’s spectrophotometric method [93]. The assay was performed in triplicated 96-well plates by adding 25 µL of 1 mM ATCI, which was used as the substrate in the assay; 125 µL of 1 mM DTNB; 25 µL of 0.1 M phosphate buffer at pH 7.4; and 25 µL of the sample supernatant. The absorbance changes at 405 nm were detected every 30 s over a period of 5 min using a microplate reader. The enzyme activity and the percent inhibition were determined.

The inhibitory activity was calculated from the enzymatic activity using this equation:

Percentage inhibition (%inhibition) = [(enzymatic activity of enzyme − enzymatic activity of a sample)/(enzymatic activity of enzyme − enzymatic activity of control)] × 100

### 4.5. Acute and Subchronic Toxicological Evaluation

The KBD formula was evaluated for its acute and subchronic toxicities according to the Organization for Economic Cooperation and Development (OECD) Guideline for the Testing of Chemicals No. 423 and No. 408, respectively. All animal studies were approved by the National Laboratory Animal Center-Animal Care and Use Committee, Mahidol University, Thailand (record no. RA2019-18 and RA2019-19 for acute and sub-chronic toxicities, respectively). Wistar rats aged 6–8 weeks and weighing 180–220 g were obtained from the National Laboratory Animal Centre, Nakorn Pathom, Thailand. All animals were quarantined for 1 day prior to maintenance in an animal cage. Animals were housed in colony cages (5 rats per cage), under standard laboratory conditions (ventilated room, 25 °C, 75% humidity, 12 h light/dark cycle) and had free access to food and water.

#### 4.5.1. Acute Oral Toxicity Study

After acclimating for 5 days, rats were fasted overnight (15–18 h) with free access to water prior to administration by oral gavage of a single dose (300 or 2000 mg/kg, 6 rats/group) of KBD powder suspended in distilled water. The clinical signs of toxicity were continuously monitored at 0.5, 4, 8, and 24 h after the treatment and daily thereafter up to 14 days. Rats were monitored for mortality, any changes in food and water consumption, and any additional clinical or behavior signs of toxicity (appearance: fur, skin, eyes, pupil size, lacrimation, piloerection, and occurrence of secretion and excretion; body function: respiration, mucous membranes, and lymph nodes; and behaviors: social interaction, posture mobility, and activity). Animals were weighed prior to treatment (day 0) and on days 7 and 14.

#### 4.5.2. Subchronic Oral Toxicity Study in Rats

Animals were randomly divided into 5 groups (I–V) of 20 each (10 females and 10 males). The KBD powder, suspended in distilled water, was administered by daily oral gavage for 90 days, to groups I–V (doses of 0, 125 (low dose), 250 (medium dose), 500 (high dose), and 500 (recovery group) mg/kg, respectively). The KBD doses were assigned based on the clinical dose (2000 mg/day) that is prescribed in the hospital [30]. This dose was converted into the appropriate dose for rats, according to the following equation: human equivalent dose (HED, mg/kg) = rat dose (mg/kg) × (rat Km/human Km), where Km is the correction factor [85]. After 90 days of treatment, rats in group V (recovery group) were continuously observed for a further 14 days after withdrawing the drug to identify the convalescence of toxicity. The animals were observed for toxicological signs (appearance: fur, skin, eyes, pupil size, lacrimation, piloerection, and occurrence of secretion and excretion; body function: respiration, mucous membranes, and lymph nodes; and behaviors: social interaction, posture mobility, and activity) and mortality throughout the experimental period. The body weight (BW), and water and food consumption were recorded weekly (on day 0 and in weekly intervals).

##### Pathological Examination

At the end of the 90-day treatment and recovery period, rats were sacrificed by decapitation under anesthesia (thiopental 50 mg/kg). Blood samples were collected with and without anticoagulant (EDTA) for hematological and biochemical studies, respectively. The organs (brain, thymus, heart, lung, liver, spleen, kidneys, adrenal glands, testis, epididymis, ovaries, and uterus) were isolated and weighed. The relative organ weight (weight of the organ as a proportion of the total body weight of each rat) was calculated and compared with the value of the control. Vital organ liver was fixed in 10% formalin for histopathological examination. All organs and tissues were embedded in paraffin and sectioned. After being stained with hematoxylin and eosin, the slides were examined with a microscope to detect any lesions.

##### Measurement of Hematological and Biochemical Parameters in Rats

For hematological examination, red blood cell (RBC), hemoglobin (HGB), hematocrit (HCT), mean corpuscular volume (MCV), mean corpuscular hemoglobin (MCH), mean corpuscular hemoglobin concentration (MCHC), red distribution width (RDW), reticulocyte (RET), platelet count (PLT), platelet distribution width (PDW), mean platelet volume (MPV), white blood cell (WBC), neutrophil (NEU), lymphocyte (LYMP), monocyte (MONO), eosinophil (EO), and basophil (BASO) measures were determined using a hematology analyzer (MEK-6318K, Nihon Kohden Co. Ltd, Bangkok, Thailand.).

For biochemical analysis, blood without additive was centrifuged at 3000× *g* at 4 °C for 10 min. Serum was separated and stored at −20 °C until the determination of levels for glucose (GLU), blood urea nitrogen (BUN), creatinine (CREA), uric acid (UA), total cholesterol (CHOL), triglyceride (TG), aspartate amino transferase (AST), alanine amino transferase (ALT), alkaline phosphatase (ALP), total protein (TP), albumin (ALB), and globulin (GLO).

### 4.6. Statistical Analyses

The results are expressed as mean ± SD and mean ± SEM for in vitro and in vivo experiments, respectively. Statistical significance was determined by one-way analysis of variance (ANOVA). For all statistical analysis, significance levels were set at *p* < 0.05.

## 5. Conclusions

KBD, which comprises parts of three different kinds of single herbal plants—*Nelumbo nucifera* Gaertn. petals, *Piper nigrum* L. fruits, and the aerial part of *Centella asiatica* (L.) Urb. —has traditionally been used as an herbal medicine in Thailand. Our previous in vitro study illustrated that KBD demonstrates multiple modes of action against the AD pathological cascade, including antioxidant, anti-AChE, anti-Aβ-aggregating, neuroprotective, and anti-apoptotic activities. The main purpose of the present study was to determine the protective effect and mechanism of KBD in Aβ_1–42_-induced AD rats and its toxicity profiles. Our results suggest that the KBD formula improved short- and long term-memory performance, as assessed using the Morris Water Maze and object recognition tasks, and that this improvement occurred through the attenuation of oxidative stress and the inhibition of AChE activity in the rat brain. Overall, the results indicate that KBD demonstrates a broader mode of action against AD compared to various classical agents. Due to the multifactorial pathogenesis of AD, KBD—which demonstrates multiple modes of action—is likely to possess greater potential for AD treatment. Furthermore, to provide safety information, the present study also assessed the acute and subchronic toxicities of KBD in rats. The acute toxicity test revealed that the limit dose of 2000 mg/kg did not cause any mortality or symptoms of toxicity. The oral subchronic toxicity assessment of KBD at doses of 125, 250, and 500 mg/kg body weight/day for 90 days revealed no adverse effects on behavior, mortality, hematology or serum biochemistry. Our investigations indicate that KBD is a nontoxic traditional medicine that has potential for AD treatment. Thus, KBD could be used as a novel alternative choice for the prevention and treatment of AD.

## Figures and Tables

**Table 1:** Hematologic parameters in Wistar rats after 90 days of treatment with KBD. Data are presented as mean ± SEM for *n* = 10.

Parameters	Treatment Groups									
	Control		125 mg/kg		250 mg/kg		500 mg/kg		Recovery	
	Males	Females	Males	Females	Males	Females	Males	Females	Males	Females
RBC, 10^6^/µL	9.99 ± 0.47	9.47 ± 0.28	9.88 ± 0.44	9.26 ± 0.33	9.83 ± 0.32	9.39 ± 0.25	9.76 ± 0.58	9.54 ± 0.42	9.68 ± 0.33	9.13 ± 0.44
HGB, g/dL	17.38 ± 0.53	17.96 ± 0.42	17.69 ± 0.71	17.50 ± 0.71	17.36 ± 0.59	17.55 ± 0.50	17.30 ± 0.91	18.01 ± 0.81	17.60 ± 0.39	17.04 ± 0.64
HCT, %	59.94 ± 1.78	57.46 ± 1.64	56.90 ± 2.32	56.11 ± 2.42	55.06 ± 2.14	55.71 ± 1.72	55.30 ± 3.19	57.56 ± 2.88	55.82 ± 1.38	53.68 ± 2.19
MCV, fl	55.04 ± 1.58	60.67 ± 1.11	57.62 ± 1.52	60.61 ± 0.77	56.01 ± 1.21	59.31 ± 1.09	56.70 ± 1.19	60.33 ± 1.16	57.69 ± 1.68	58.86 ± 1.46
MCH, pg	17.41 ± 0.47	18.97 ± 0.29	17.91 ± 0.45	18.91 ± 0.29	17.65 ± 0.40	18.70 ± 0.35	17.73 ± 0.41	18.88 ± 0.34	18.18 ± 0.54	18.68 ± 0.35
MCHC, g/dL	31.62 ± 0.30	31.26 ± 0.27	31.08 ± 0.23	31.19 ± 0.36	31.53 ± 0.33	31.51 ± 0.20	31.30 ± 0.35	31.28 ± 0.27	31.52 ± 0.33	31.74 ± 0.28
RDW, fl	32.9 ± 1.10	30.54 ± 0.73	34.1 ± 1.16	30.08 ± 0.68	33.19 ± 0.68	29.38 ± 0.59	33.30 ± 1.31	29.59 ± 0.55	33.20 ± 0.65	29.15 ± 0.48
RET, 10^3^/µL	289.57 ± 19.1	323.21 ± 38.98	304.65 ± 32.8	306.61 ± 45.08	297.02 ± 33.1	297.93 ± 37.1	279.96 ± 21.1	324.78 ± 39.57	306.92 ± 28.1	293.44 ± 50.5
PLT, 10^3^/µL	833.60 ± 81.4	757.60 ± 56.51	847.70 ± 105	770.70 ± 73.14	772.80 ± 63.7	800.40 ± 57.3	838.00 ± 85.3	838.90 ± 115.4	796.30 ± 62.5	842.50 ± 67.4
PDW, fl	8.20 ± 0.35	7.97 ± 0.36	8.32 ± 0.36	8.03 ± 0.22	8.00 ± 0.39	8.15 ± 0.31	8.69 ± 0.52	8.37 ± 0.35	8.91 ± 0.33	8.15 ± 0.30
MPV, fl	6.94 ± 0.24	7.01 ± 0.24	7.13 ± 0.19	7.02 ± 0.14	6.83 ± 0.26	6.99 ± 0.20	7.29 ± 0.30	7.16 ± 0.13	7.54 ± 0.18	7.05 ± 0.20
WBC, 10^3^/µL	7.94 ± 0.97	6.92 ± 1.42	9.78 ± 1.33	6.21 ± 0.89	7.58 ± 1.29	5.63 ± 1.32	7.23 ± 2.53	7.14 ± 1.21	8.78 ± 1.30	6.65 ± 1.08
NEU, 10^3^/µL	0.87 ± 0.15	0.47 ± 0.18	0.90 ± 0.42	0.26 ± 0.16	0.73 ± 0.42	0.46 ± 0.25	0.55 ± 0.36	0.42 ± 0.16	1.01 ± 0.15	0.48 ± 0.08
LYMPH,10^3^/µL	6.58 ± 0.83	6.04 ± 1.20	8.35 ± 1.44	5.65 ± 0.82	6.27 ± 1.33	4.83 ± 1.07	6.22 ± 2.46	6.32 ± 1.07	7.17 ± 1.14	5.81 ± 1.00
MONO,10^3^/µL	0.41 ± 0.18	0.36 ± 0.12	0.45 ± 0.12	0.27 ± 0.05	0.46 ± 0.12	0.30 ± 0.09	0..38 ± 0.16	0.35 ± 0.07	0.48 ± 0.11	0.31 ± 0.06
EO, 10^3^/µL	0.07 ± 0.03	0.05 ± 0.03	0.07 ± 0.03	0.03 ± 0.01	0.10 ± 0.03	0.04 ± 0.02	0.07 ± 0.02	0.04 ± 0.01	0.10 ± 0.02	0.04 ± 0.02
BASO, 10^3^/µL	0.01 ± 0.01	0.01 ± 0.01	0.02 ± 0.02	0.01 ± 0.01	0.02 ± 0.01	0.01 ± 0.01	0.01 ± 0.01	0.01 ± 0.01	0.01 ± 0.01	0.01 ± 0.01

**Table 2:** Serum biochemical parameters in Wistar rats after 90 days of treatment with KBD. Data are presented as mean ± SEM for *n* = 10.

Parameters	Treatment Groups									
	Control		125 mg/kg		250 mg/kg		500 mg/kg		Recovery	
	Males	Females	Males	Females	Males	Females	Males	Females	Males	Females
GLU	299.7 ± 72.3	148.8 ± 73.7	350.5 ± 43.1	172.7 ± 97.5	318.5 ± 57.5	150.4 ± 45.6	324.2 ± 66.2	186.9 ± 69.8	396.1 ± 57.8	148.3 ± 60.7
BUN	21.1 ± 1.7	21.0 ± 2.7	21.2 ± 1.3	21.1 ± 1.8	21.7 ± 2.5	22.4 ± 2.7	23.0 ± 2.0	23.0 ± 2.8	24.4 ± 2.9	23.2 ± 4.3
CREA	0.4 ± 0.0	0.5 ± 0.1	0.4 ± 0.0	0.5 ± 0.0	0.4 ± 0.0	0.5 ± 0.0	0.4 ± 0.0	0.4 ± 0.0	0.5 ± 0.0	0.5 ± 0.1
UA	6.9 ± 1.4	4.1 ± 0.8	7.6 ± 0.8	4.3 ± 0.9	7.6 ± 1.3	3.9 ± 0.3	6.7 ± 1.3	4.5 ± 1.1	8.7 ± 1.0	4.5 ± 0.4
CHOL	87.9 ± 17.8	99.6 ± 15.9	87.7 ± 7.6	104.7 ± 15.4	89.0 ± 11.3	107.2 ± 12.6	93.6 ± 11.4	117.9 ± 14.1	94.6 ± 22.0	106.6 ± 20.2
TG	110.2 ± 21.7	68.8 ± 15.2	111.9 ± 28.3	78.5 ± 16.8	128.7 ± 34.4	78.8 ± 20.1	128.5 ± 29.8	103.0 ± 32.0	143.5 ± 29.8	76.1 ± 19.2
AST	99.7 ± 16.2	99.3 ± 17.1	100.1 ± 23.4	90.5 ± 9.7	110.6 ± 28.2	96.7 ± 9.8	94.8 ± 16.4	96.3 ± 11.0	157.6 ± 39.5	102.4 ± 10.0
ALT	54.6 ± 14.2	38.9 ± 4.4	61.9 ± 21.5	38.3 ± 4.3	70.9 ± 25.0	44.9 ± 8.0	57.3 ± 22.7	40.5 ± 4.5	101.4 ± 30.4	48.7 ± 3.6
ALP	66.6 ± 5.3	32.8 ± 3.3	69.3 ± 10.8	33.0 ± 3.2	69.9 ± 11.7	35.2 ± 0.2	68.0 ± 8.0	33.0 ± 4.0	73.8 ± 8.9	32.1 ± 6.5
TP	6.9 ± 0.2	7.3 ± 0.2	7.3 ± 0.2	7.3 ± 0.3	7.2 ± 0.3	7.2 ± 0.2	7.4 ± 0.7	7.5 ± 0.3	8.3 ± 0.5	8.0 ± 0.5
ALB	5.3 ± 0.2	5.9 ± 0.1	5.6 ± 0.2	5.7 ± 0.1	5.4 ± 0.2	5.6 ± 0.2	5.4 ± 0.2	5.5 ± 0.3	5.7 ± 0.2	5.8 ± 0.2
GLO	1.7 ± 0.1	1.4 ± 0.1	1.7 ± 0.3	1.4 ± 0.2	1.8 ± 0.2	1.6 ± 0.3	1.8 ± 0.3	1.8 ± 0.2	2.5 ± 0.4	2.1 ± 0.3

**Table 3:** The changes in the relative organ weight for subchronic administration of KBD in female and male rats.

Parameters	Treatment Groups									
	Control		125 mg/kg		250 mg/kg		500 mg/kg		Recovery	
	Males	Females	Males	Females	Males	Females	Males	Females	Males	Females
Adrenal GI (L)	0.0448 ± 0.0024	0.0476 ± 0.0057	0.0475 ± 0.0062	0.0492 ± 0.0039	0.044 ± 0.0035	0.0488 ± 0.0033	0.0431 ± 0.0044	0.047 ± 0.0037	0.0413 ± 0.0033	0.0451 ± 0.0041
Adrenal GI (R)	0.0394 ± 0.0031	0.0431 ± 0.0030	0.0414 ± 0.0050	0.044 ± 0.0051	0.0377 ± 0.0029	0.0441 ± 0.0068	0.0356 ± 0.0031	0.0432 ± 0.0055	0.0372 ± 0.0043	0.0404 ± 0.0036
Epididymis/ Ovary (L)	0.6055 ± 0.0853	0.0498 ± 0.0059	0.6107 ± 0.0589	0.0483 ± 0.0082	0.5919 ± 0.0691	0.0507 ± 0.0079	0.5995 ± 0.0216	0.0539 ± 0.0106	0.5239 ± 0.0582	0.0478 ± 0.0042
Epididymis/ Ovary(R)	0.6041 ± 0.0421	0.0508 ± 0.0051	0.5953 ± 0.0935	0.0483 ± 0.0059	0.5788 ± 0.0405	0.0526 ± 0.0090	0.5977 ± 0.0272	0.0556 ± 0.0120	0.5197 ± 0.0309	0.0461 ± 0.0083
Thymus	0.3293 ± 0.0415	0.2449 ± 0.0523	0.3189 ± 0.0605	0.265 ± 0.0460	0.3198 ± 0.0511	0.2689 ± 0.0679	0.3139 ± 0.0388	0.2553 ± 0.0596	0.3113 ± 0.0424	0.2441 ± 0.0374
Spleen	0.9386 ± 0.1238	0.6466 ± 0.0479	0.9613 ± 0.0877	0.6531 ± 0.0456	0.9154 ± 0.0966	0.6713 ± 0.0617	0.9475 ± 0.0787	0.6787 ± 0.0659	0.9126 ± 0.0546	0.5612 ± 0.1796
Uterus	-	0.596 ± 0.1914	-	0.5619 ± 0.2389	-	0.6254 ± 0.2990	-	0.7185 ± 0.2096	-	0.4648 ± 0.1352
Heart	1.4229 ± 0.0938	0.9203 ± 0.0617	1.4563 ± 0.1552	0.935 ± 0.0590	1.3984 ± 0.0730	0.9209 ± 0.0554	1.3681 ± 0.0731	0.9121 ± 0.0591	1.5039 ± 0.0481	0.9422 ± 0.0445
Kidney (L)	1.2634 ± 0.0697	0.7417 ± 0.0377	1.2676 ± 0.1247	0.7477 ± 0.0309	1.1942 ± 0.0917	0.7392 ± 0.0307	1.2238 ± 0.0941	0.7161 ± 0.1934	1.2276 ± 0.0823	0.7489 ± 0.0447
Kidney (R)	1.2944 ± 0.0842	0.7816 ± 0.0465	1.2705 ± 0.1355	0.778 ± 0.0545	1.2197 ± 0.0874	0.7655 ± 0.0328	1.2338 ± 0.1104	0.8047 ± 0.0681	1.2604 ± 0.0750	0.781 ± 0.0427
Testis (L)	1.8789 ± 0.1077	-	1.9031 ± 0.1869	-	1.8457 ± 0.1417	-	1.8802 ± 0.1077	-	1.8824 ± 0.0711	-
Testis (R)	1.8803 ± 0.0737	-	1.874 ± 0.1740	-	1.7906 ± 0.1309	-	1.8368 ± 0.1234	-	1.8592 ± 0.0967	-
Brain	2.2057 ± 0.0527	1.9893 ± 0.1008	2.1515 ± 0.0603	2.0147 ± 0.0483	2.1639 ± 0.0836	1.9874 ± 0.0633	2.19 ± 0.0792	2.0344 ± 0.0578	2.2028 ± 0.0779	2.0501 ± 0.0385
Liver	13.2839 ± 0.9141	7.1394 ± 0.5813	13.7123 ± 1.6271	6.7335 ± 1.8537	13.0735 ± 0.6401	7.113 ± 0.6302	13.698 ± 0.9184	7.6398 ± 0.9619	13.3958 ± 0.8096	7.0372 ± 0.7335

## Data Availability

The data is contained within the article.
